# How Cabbage Aphids *Brevicoryne brassicae* (L.) Make a Choice to Feed on *Brassica napus* Cultivars

**DOI:** 10.3390/insects10030075

**Published:** 2019-03-15

**Authors:** Zhong-Ping Hao, Hai-Xia Zhan, Yu-Long Wang, Shu-Min Hou

**Affiliations:** National Oil Crops Improvement Center, Hefei Rapeseed Subcenter, Crop Research Institute, Anhui Academy of Agricultural Sciences, Hefei 230031, China; zhanertu@163.com (H.-X.Z.); yulong4444@126.com (Y.-L.W.)

**Keywords:** cabbage aphids, electrical penetration graph, feeding preference, oilseed rape, probing behavior, resistance to aphids

## Abstract

Plant resistance to aphids might be present in different plant tissues, such as the epidermis, mesophyll and phloem, but not all of them play a key role in determining the feeding preference of aphids. In this study, electrically recorded feeding behaviors of cabbage aphids were combined with choice tests and microscopic observations to understand the feeding preference of cabbage aphids on oilseed rape cultivars. The choice tests showed that more cabbage aphids survived on ‘Qianyou18’, and less on ‘Zhongshuang11’, compared with the other cultivars. The results of the choice tests were paradoxical with the results analyzed from the general and mesophyll-associated variables. The thick upper epidermis with bushy long trichomes on the leaves of ‘Zhongshuang11’ delayed the first probe of the cabbage aphids. The duration of phloem-feeding was similar among the four cultivars although there were differences in the hindrance of the mesophyll. However, salivation was increased when the aphids fed on ‘Zhongshuang11’, further indicating that the leaf’s physical properties could be important for aphid feeding preference on the four cultivars.

## 1. Introduction

Aphids, as plant phloem sap feeders, insert their stylets into the plant tissue, covering the distance from the epidermis to the phloem vessel, and feed on substances in the sieve elements [1,2]. Meanwhile, plants have developed a wide variety of physical and biochemical defense mechanisms against aphid feeding [3] at different layers of tissues (surface, epidermis, mesophyll tissues and/or phloem elements). The wide variety of defense mechanisms in plants vary at different levels, depending on the aphid and plant species/cultivars. To maximize survival and reproduction, it is necessary for aphids to have efficient countermeasures to locate and exploit the host plant resistance. Generally, the response of aphids to stimuli necessary for host-plant discrimination can reflect the aphid feeding preference [4]. The effects of various plant species/cultivars on the fitness and abundance of aphids have been studied extensively, but the resistance location and the mechanisms of resistance vary in different aphid-plant systems [5,6,7,8]. The acceptance of host plants by aphids occurs stepwise through behavioral sequences to sustained feeding [9]. On the leaf surface, the structure and the physical properties could affect host selection and colonization of aphids significantly [10]. Aphids can feel these leaf properties by vision, olfactory, gustatory and tactile sensations [4]. Especially, the role of glandular trichomes in the defense against insects is well documented, and it varies with plant accessions and cultural conditions [11,12,13]. In epidermis and mesophyll probing, any obstacles could impede localizing or reaching phloem vessels by the stylets [7,14], such as a chemical factor (possible role of hydroxamic acids) outside the phloem in the case of barley resistant to *Rhopalosiphum padi* (Linnaeus) [15], and a mechanical barrier (e.g. pectin composition) outside the sieve elements in the cases of sorghum resistant to *Schizaphis graminum* (Rondani) [16] and of wheat resistant to *R. padi* (L.) [17]. Phloem resistance interferes with the initiation of a sap ingestion event and hinders ingestion, and has been reported for several aphid-plant interactions [6,18,19,20]. It seems that aphids are, to some extent, dependent on host-plant specific resistance at different layers of tissues to distinguish between suitable and unsuitable host plants [4].

For cabbage aphids, *Brevicoryne brassicae* (L.) (Hemiptera: Aphididae), Gabrys and Pawluk (1999) [21] studied the effects of several plant species that represented various levels of acceptability by the cabbage aphids: *Sinapis alba* (L.) as a permanent host plant, *Capsella bursa-pastoris* (L.) (Medikus), *Thlaspi arvense* (L.), *Lunaria annua* (L.), *Erysimum cheiranthoides* (L.) as accidental host plants, and *Vicia faba* (L.) as a non-host plant. They found that the mechanisms of plant resistance to the cabbage aphids varied among the plant species. For *S. alba*, aphid probing and sap ingestion were rarely interrupted. For *C. bursa-pastoris* and *T. arvense*, a considerable delay between finding and accepting the phloem, and an increased proportion of salivation time were detected. For *L. annua* and *E. cheiranthoides*, stylet penetration was deterred in the peripheral tissues, and the periods of sap ingestion in the phloem were short or did not occur, but the salivation predominated. On *V. faba*, *B. brassicae* did not show any penetration into the phloem vessels.

On three cultivars of oilseed rape *Brassica napus* (L.) with different resistance levels against cabbage aphids, Hao et al. (2017) [22] showed that the aphids delayed their first probe, had longer pathway durations and shorter ingestion durations on the resistant ‘Qinyou79’, compared with the susceptible ‘Qinyou10’. This study focused on the establishment of a rapid screening method for the resistance of oilseed rape cultivars to aphids, but ignored the mechanism of resistance to aphid feeding. Thus, we conducted choice tests in greenhouse, microscopic observations, and electropenetrography (EPG) experiments to study the mechanisms utilized by oilseed rape plants resistant to cabbage aphids, and aphid feeding preference.

## 2. Materials and Methods 

### 2.1. Aphids and Plants Rearing

Cabbage aphids, *Brevicoryne brassicae*, were collected from greenhouse grown oilseed rape at the Institute of Vegetables, Zhejiang Academy of Agricultural Sciences. In order to avoid a behavioral bias towards susceptible oilseed rape cultivars, the aphids were reared in cages of a climate chamber over one year on a *Brassica oleracea* var. *capitata* (L.) cultivar at 25 ± 1 °C, 75 ± 5% RH and 16:8 (L:D) photoperiod. Thus, the aphids could not adapt to any of the oilseed rape cultivars used. All aphids used in the experiments came from a newly established clone of *B. brassicae* from a single virginoparous apterous individual. Recently molted alate adults were collected for later choice tests and recently molted (2 days) apterous adults were used for later EPG monitoring tests.

Four *Brassica napus* var. *napus* (L.) cultivars, including ‘Qianyou18’, ‘Zhongheza488’, ‘Heyou202’, and ‘Zhongshuang11’, were chosen from the collection maintained at the Laboratory of Plant Breeding (Anhui Academy of Agricultural Sciences, China). In a climate room, plants were grown in plastic pots (13-cm diameter) with a mixture of peat moss, vermiculite, organic fertilizer (N + P_2_O_5_ + K_2_O ≥ 2%, organic matter ≥ 40%, Zhongnuo, Huaian, Jiangsu, China), and perlite (10:10:10:1 ratio) under 25 ± 1 °C, 75 ± 5% RH and 12:12 (L:D) photoperiod, and watered regularly, without additional fertilizer added. Plants of each *B. napus* cultivar with four fully expanded leaves were used in EPG experiments according to our previous study [22].

### 2.2. Investigation of Aphid Population in Greenhouse

A choice test was created to investigate the feeding preference of cabbage aphids. In a well-ventilated greenhouse of Zhejiang Academy of Agricultural Sciences, an area of 6 m × 12 m was roughly divided into four equal rectangle sections (3 m × 6 m) without any physical separation, each of which was planted in September with one cultivar (50 cm row spacing, 10 cm plant spacing). Without any chemical treatment, the cultivation and management measures including watering and fertilizing were the same as those in the field. One hundred alate adult aphids were placed in the central five plants of each cultivar (around the intersection of rectangular diagonals) at the two-leaf stage. A regular monthly survey of the aphid population was carried out by counting the numbers of nymphs and adults on the plants from November in 2016 to March in 2017 in the greenhouse. The count of the aphid population (adults and nymphs) was recorded by sampling 10 plants of each cultivar at random.

### 2.3. Microscopic Observation of Leaf Surface

According to the methods of Yan and Wang (2017) [23] with minor modifications, the uppermost first leaf of each cultivar at the four-leaf stage was selected and prefixed with 2% glutaraldehyde overnight. The next morning, the leaf was washed in a phosphate buffer (PBS, pH = 7.4), then postfixed in 2% OsO4 for 1 h, and washed in PBS at pH 7.4 again. In the end, the preparations were dehydrated in a graded alcohol series, followed by embedding in Epon 812 (SPI Supplies, Structure Probe, Inc., West Chester, The United States of America). Then a semi-thin section (3–4 µm) was performed and examined using an inverted phase contrast microscope (Leica DM IRB, Leica Microsystems, Wetzlar, Germany). The thickness of the upper epidermis was measured, and the number and the length of trichomes were recorded. Ten leaves per replicate were observed and three replicates were carried out for each cultivar.

### 2.4. EPG Experiments

Aphid behavior was monitored using the EPG technique as described by Hao et al. (2017) [22] with minor modifications. In the EPG experiments, aphids and plants need to be connected to form a closed circuit during aphid feeding. For this connection, a gold wire is glued to the aphid and a copper electrode is inserted into the soil near the roots of a potted plant. When the aphid stylet penetrates the plant, a closed circuit is created [3,24,25]. The aphid electrode was connected to a four-channel DC-EPG system (Giga-4; EPG Systems, Wageningen, The Netherlands) and the EPG output was recorded with PROBE 3.5 (hardware and software from EPG-Systems, Wageningen, The Netherlands). Inside a Faraday cage, the tethered aphid was rapidly (<30 min after collecting from the rearing plant) placed on the upper side of mature leaf midrib of the test plant, from which a copper electrode was connected and inserted in the soil. Aphids and plants were used only once for each recording. The feeding behavior of *B. brassicae* was monitored for 6 h. Continuous records from 30 individuals were conducted in laboratory conditions under constant lighting and at 25 ± 1 °C. The EPG profiles were recorded by A/D card (DI-710 format, Dataq Instruments Incorporated, The United States of America) and analyzed by the Stylet^+^ software. The definitions of waveforms scored in EPG analyses for each tested aphid were listed in Table 1, and the data were automatically analyzed using the MS Excel workbook for the automatic parameter calculation of EPG data (version 4.4) developed by Sarria et al. (2009) [26].

### 2.5. Statistical Analysis

The EPG data were transformed by square-root for frequency variables, natural log for time variables, and square arcsine for percentage variables [22]. The data were statistically analyzed using the analysis of variance (One-Way ANOVA) followed by the unrestricted least significant differences (LSD) procedure in the SAS 9.2 software (SAS Institute Inc., Cary, NC, The United States of America). The level for significance was set to *P* < 0.05.

## 3. Results

### 3.1. Aphid Performance in Greenhouse

**Figure 1:**
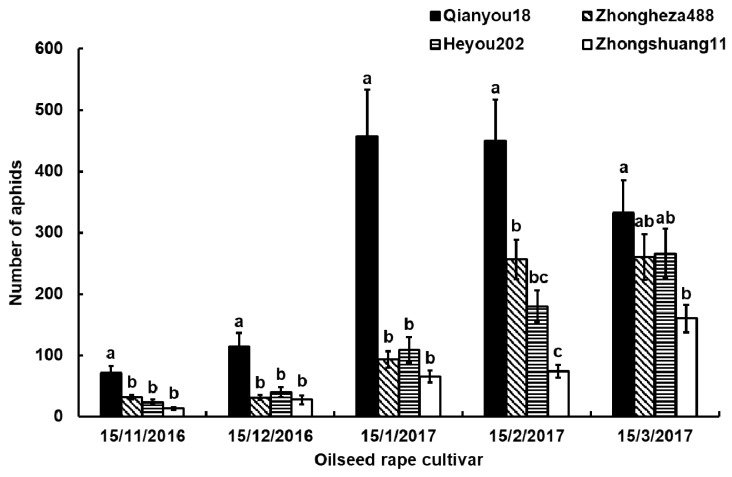
The dynamics of cabbage aphids on four oilseed rape cultivars in a greenhouse. Values are means ± standard error of mean(SEM) of the number of aphids (adults and nymphs). Data were analyzed by analysis of variance (ANOVA) followed by the unrestricted least significant differences (LSD) test. The significance level was set at *P* < 0.05. Bars represent stand error (SE). The same lowercase letters above columns at the same investigation time represent no significant differences among the cultivars.

The monthly records of the aphid population on each cultivar are given in Figure 1. The cabbage aphids could survive and develop on all four cultivars. More aphids developed on ‘Qianyou18’ and fewer aphids on ‘Zhongshuang11’. ‘Zhongheza488’ and ‘Heyou202’ had intermediate levels of infestation. The aphid numbers on ‘Qianyou18’ were the highest with peaks at the third and fourth months. After five months, no statistical difference was detected among ‘Qianyou18’, ‘Zhongheza488’ and ‘Heyou202’.

### 3.2. Leaf Surface Characteristic

**Figure 2:**
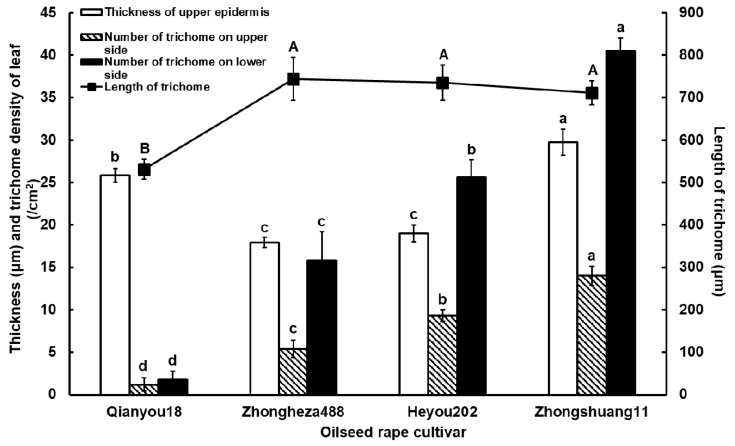
The leaf surface characteristics of four oilseed rape cultivars. Data were analyzed by ANOVA followed by the unrestricted least significant differences (LSD) test. The significance level was set at *P* < 0.05. Bars represent stand error (SE). The same lowercase letters above the columns of the same color represent no significant differences among the tested cultivars, and the same uppercase letters above the black squares represent no significant differences among the tested cultivars.

There were significant differences in leaf surface characteristics among the four cultivars, as shown in Figure 2. ‘Zhongshuang11’ had the thickest upper epidermis (29.75 ± 1.51 µm) among the four cultivars. The epidermis thickness of ‘Qianyou18’ (25.87 ± 0.81 µm) was thinner than that of ‘Zhongshuang11’, but significantly thicker than that of ‘Zhongheza488’ (17.94 ± 0.61 µm) and ‘Heyou202’ (19.00 ± 0.97 µm) (*F*_3,55_ = 32.1801, *P* < 0.01). However, ‘Qianyou18’ had significantly fewer trichomes on the whole surface of its leaf than ‘Zhongshuang11’ which possessed denser trichomes than other two cultivars (*F*_3,18_ = 33.7505, *P* < 0.01 on the upper side; *F*_3,15_ = 35.0661, *P* < 0.01 on the lower side). The length of the trichomes on ‘Qianyou18’ was 530.98 ± 23.21 μm, which was significantly shorter than those of the other cultivars (*F*_3,40_ = 5.1259, *P* = 0.0043).

### 3.3. Aphid Probing and Feeding Behavior

EPG recordings were conducted for the cabbage aphids on each of the four cultivars to understand the aphid feeding preference. Variables derived from analysis of EPG waveforms were used to assess aphid behavior in specific plant tissues (Table 1).

As shown in Table 2, most of the general variables had significant differences among the four cultivars. Compared with aphids on other cultivars, the cabbage aphids on ‘Qianyou18’ spent a shorter time to complete probing and engaged more in non-probing behaviors, e.g., walking. The non-probing duration and the time to first phloem contact were longer on ‘Qianyou18’ and ‘Zhongshuang11’ than those on ‘Zhongheza488’.

**Figure 3:**
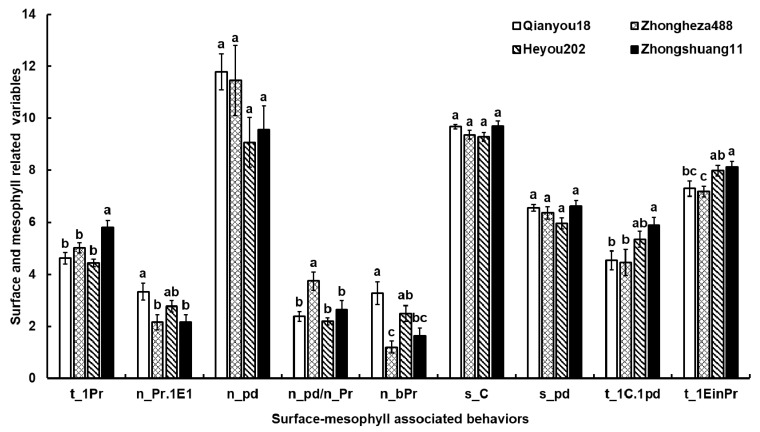
The electropenetrography (EPG) variables of the *B. brassicae* stylet pathway before reaching the phloem tissue of the host plants. Bars represent stand error (SE). The data were compared using the analysis of variance (ANOVA) followed by the unrestricted least significant differences (LSD) after the square-root transformation for frequency variables and natural log transformation for time variables. The level for significance was set to *P* < 0.05. Different lowercase letters on the columns indicated the significant differences among the four cultivars.

In surface-related variables, superficial probes were found on ‘Zhongshuang11’ significantly later than when compared with those on other cultivars (*F*_3,40_ = 8.3040, *P* = 0.0002) (Figure 3). 

In the mesophyll, cabbage aphids made significantly more penetrations and more (two-fold) short probes on ‘Qianyou18’ than on ‘Zhongheza488’ and ‘Zhongshuang11’ (*F*_3,41_ = 7.1144, *P* = 0.0006). The aphids on ‘Qianyou18’ and ‘Zhongheza488’ started penetrating the mesophyll cells more quickly and reached the phloem sooner than those on ‘Zhongshuang11’. In addition, the aphids penetrated more cells per probe on ‘Zhongheza488’ than on other cultivars. The other mesophyll-related variables had no significant differences among the four cultivars (Figure 3).

In the phloem, as measured by phloem-related variables, cabbage aphids showed similar ingestion behavior among the four cultivars (Table 3). However, the aphids secreted more saliva in the phloem of ‘Zhongshuang11’ than in the phloem of ‘Qianyou18’ and other cultivars (*F*_3, 34_ = 4.3705, *P* = 0.0105), and injected the least saliva followed by sustained ingestion on ‘Zhongheza488’ (*F*_3, 29_ = 4.3988, *P* = 0.0114).

### 3.4. Relative Average Duration of Main Waveforms over 6 h

**Figure 4:**
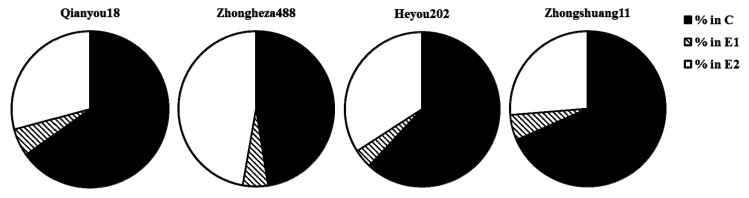
The percentages of different activities in relation to the complete probing of *B. brassicae* on oilseed rape during 6-hour electropenetrography (EPG) experiments. The % in C represents the percentage of probing spent in the pathway; the % in E1 represents the percentage of probing spent in salivation; the % in E2 represents the percentage of probing spent in sap ingestion.

As shown in Figure 4, the aphids ingested less sap, and spent longer pathway durations on ‘Zhongshuang11’ than on other three cultivars, but no statistical differences were detected in the percentages of these steps among the four cultivars.

## 4. Discussion

Cabbage aphids could survive and develop on all four cultivars, but on ‘Qianyou18’, they developed better than on other three cultivars based on the population (Figure 1). This implies that the four cultivars have different attractions or suitabilities to the cabbage aphids, rather than antibiosis. ‘Qianyou18’ seemed to be more attractive or suitable to the cabbage aphids than other three cultivars in the greenhouse. More aphids on ‘Qianyou18’ might come from other cultivars (attractive). Likewise, the reproductive capacity of aphids on this cultivar might be higher (suitable). We conducted microscopic observations and EPG experiments to discriminate between these two reasons.

A considerable delay in initiating the first probing on the leaf surface of ‘Zhongshuang11’ was observed, compared to other cultivars (Figure 3). The long time to the first probe from the start of EPG mainly reflects the effects of mechanical stimuli on the leaf surface, such as the presence of trichomes and the thickness of the epidermis [18]. Our results from the microscopic observations showed that the leaves of ‘Zhongshuang11’ possessed the thickest upper epidermis and the largest number of trichomes among the four cultivars. Its trichome length was also significantly longer than that of ‘Qianyou18’. The trichome length and the number on ‘Qianyou18’ were the lowest among the four cultivars (Figure 2). These results demonstrate that trichomes play an important role in determining the aphid feeding preference for first probing and that rare short trichomes could interfere less with the aphid probing. The effects of trichome length and density and leaf thickness also have been demonstrated and are variable in other aphid-plant systems. For example, in the case of the sugarcane aphid *Melanaphis sacchari* (Zehntner), the leaves of resistant *Sorghum bicolor* (L.) (Moench) cultivars were significantly thinner and possessed fewer and longer trichomes than the leaves of susceptible cultivars [10]. In a study on the resistance of *Fragaria ananassa* (Duchesne) cultivars to aphids, the resistance of the plants was positively correlated with trichome density on the lower side of the leaves and had no obvious relationship with trichome length [27]. Alvarez et al. (2006) [5] tested trichome effects on resistance by assessing the behavior of *Myzus persicae* (Sulzer) on *Solanum berthaultii* (Hawkes) and *S. tarijense* (Hawkes) plants with and without trichomes. The plants were mechanically wiped off using a cellulose cleaning tissue under running tap water to remove all glandular parts and secretions of the trichomes. They found that the trichomes completely prevented aphids from sustained phloem-feeding, although some short periods of phloem ingestion occurred. They speculated that the glandular secretions and tarsal irritation interfered persistently with probing activities. Glandular trichome as a resistance character in tomatoes and potatoes has been used for traditional breeding for host plant resistance to aphids [28].

In the mesophyll, the total duration of the pathway (s_C) spent by the aphids was similar among the four cultivars (Figure 3), although the aphids made more penetrations during pathway on ‘Qianyou18’, and tasted more cells of ‘Zhongheza488’ than those of the other cultivars. During the short cell punctures, the maxillary stylets pierce the protoplast and the vacuole, which form the main storage sites for secondary metabolites of plants (potential allelochemicals) to obtain additional cues for phloem finding [4] and to transmit non-persistent plant viruses vectored by aphids [29]. However, owing to the similar pathway duration among the four cultivars, this suggests that the mesophyll-related hindrance might be overcome by the aphids and has little impact on the aphid feeding preference. 

After the stylets entered into the phloem, a substantial proportion of salivation time was detected in ‘Zhongshuang11’, which was significantly higher than the proportion of salivation time for the other three cultivars, and delayed the aphid ingestion (Table 3). This indicates a deterrent factor in the phloem elements that may impede aphid settling. It has been established that aphid saliva might be used to neutralize plant defense mechanisms located in sieve elements [30], such as by preventing sieve element sealing [25]. However, the duration of phloem-feeding was similar among the four cultivars, suggesting that the aphids encounter no feeding deterrents [31,32,33,34], such as low nutritional quality in the phloem sap [35,36]. During the 6-h experiments, the percentages of pathway, salivation and ingestion were not significantly different among the four cultivars (Figure 4). It is important to note that the EPG experimental set-up was of a no-choice nature (i.e., aphids were placed on the plants). Aphids might have walked away after a short probe if they were free to move [37,38]. In our choice tests, the freely moving aphids landing on ‘Zhongshuang11’ might move away to ‘Qianyou18’ after a short probe on the leaf surface of ‘Zhongshuang11’. If aphids had a rapid mechanism for rejection of unsuitable host plants, they would increase their time for reproduction [4]. It is suggested that more aphids could be attracted and reproduce faster on ‘Qianyou18’ than on ‘Zhongshuang11’ in the greenhouse.

## 5. Conclusions

In conclusion, the hindrance against cabbage aphids is located at different layers of tissues (surface, epidermis, mesophyll tissues, and/or phloem elements) in different oilseed rape cultivars. However, not all of them play a key role in determining the feeding preference of cabbage aphids. Among the oilseed rape cultivars, where the phloem sap is suitable for cabbage aphids, the factors determining the aphid feeding preference might be located at the leaf surface, such as the morphology of the trichomes. In addition, chemical cues in plant tissues also might be used to recognize the host plants by *B. brassicae* [21]. Several studies have shown that walking apterae do respond to host-plant odors in olfactometers [4,39,40,41]. Future works on the chemical cues should be conducted.

## Figures and Tables

**Table 1:** The definitions of the waveforms scored in the electropenetrography (EPG) analyses.

Acronym	Variable Type	Definition
*General*		
n_Pr	Frequency	Number of probes
s_Pr	Time	Total probing time
s_nE	Time	Total duration of the no phloematic phase
s_np	Time	Total time of the non-probing intervals
s_np.1E	Time	Duration of the nonprobe period before the 1^st^ E
t_1E2rec	Time	Time from the start of EPG to the 1^st^ E2
t_1Erec	Time	Time from the start of EPG to the 1^st^ E
*Surface-mesophyll (Leaf)*		
t_1Pr	Time	Time to the first probe from the start of EPG
n_bPr	Frequency	Number of short probes (C < 3 min)
n_Pr.1E1	Frequency	Number of probes before the 1^st^ E1 (first phloem contact)
s_C	Time	Total C duration with pd
n_pd	Frequency	Number of pd
s_pd	Time	Total duration of pd
n_pd/n_Pr	Frequency	Average number of pd per probe
t_1C.1pd	Time	Time from the beginning of the 1^st^ probe to the first pd
t_1EinPr	Time	Time from the beginning of that probe to the 1^st^ E
*Phloem*		
s_E	Time	Total duration of the E phases
n_E1	Frequency	Number of E1 periods
s_E1	Time	Total duration of E1
d_E1followedby1sE2	Time	Duration of the E1 followed by the first sustained E2 (>10 min)
s_E1followedbysE2	Time	Total duration of E1 followed by sustained E2 (>10 min)
t_endLpd.E1followedbysE2	Time	Time from the end of the last pd to the beginning of the E1 followed by the sustained E2 (>10 min)
rel_E1_E12	Index	Relative amount of E1 on E12
s_E2	Time	Total duration of E2 periods
s_longestE2	Time	Duration of the longest E2
E2index	Index	phloemian index: % of the time of the E2 after the start of the 1^st^ E2
%sE2/E2	Index	Relative amount of sE2 on E2

**Table 2:** The general variables of the *B. brassicae* feeding behavior on the four oilseed rape cultivars.

Variables ^1^	Qianyou18	Zhongheza488	Heyou202	Zhongshuang11
n_Pr	5.30 ± 0.46a	3.38 ± 0.50b	4.33 ± 0.47ab	3.67 ± 0.50b
s_Pr	10.04 ± 0.04b	10.24 ± 0.01a	10.16 ± 0.02a	10.19 ± 0.02a
s_nE	9.90 ± 0.08a	9.45 ± 0.17a	9.67 ± 0.12a	9.71 ± 0.23a
s_np	8.18 ± 0.22a	6.50 ± 0.24c	7.54 ± 0.26ab	7.24 ± 0.35bc
s_np.1E	7.22 ± 0.24a	6.04 ± 0.31b	6.88 ± 0.28ab	7.02 ± 0.34a
t_1Erec	9.08 ± 0.23a	8.40 ± 0.20b	8.81 ± 0.07ab	9.37 ± 0.22a
t_1E2rec	9.19 ± 0.24a	8.71 ± 0.22a	8.82 ± 0.07a	9.54 ± 0.24a

^1^ The values in the table show means ± stand error (SE). The data were compared using the analysis of variance (ANOVA) followed by the unrestricted least significant differences (LSD) after the square-root transformation for frequency variables, and natural log transformation for time variables. The level for significance was set to *P* < 0.05. The values followed by the same lowercase letter within a row represent no statistical differences among the four cultivars. The acronym of the variables in the first column was defined in Table 1.

**Table 3:** The phloem-related variables of the *B. brassicae* feeding on the four oilseed rape cultivars.

Variables ^1^	Qianyou18	Zhongheza488	Heyou202	Zhongshuang11
n_E1	1.70 ± 0.29a	2.20 ± 0.39a	1.16 ± 0.19a	1.42 ± 0.28a
s_E	8.75 ± 0.20a	9.24 ± 0.30a	8.74 ± 0.44a	8.29 ± 0.63a
s_E1	5.38 ± 0.22a	5.47 ± 0.41a	4.83 ± 0.22a	5.64 ± 0.29a
s_E2	8.85 ± 0.15a	9.06 ± 0.38a	8.64 ± 0.51a	8.46 ± 0.66a
s_longestE2	8.49 ± 0.22a	8.80 ± 0.45a	8.42 ± 0.50a	8.31 ± 0.66a
d_E1followedby1sE2	4.34 ± 0.04a	4.10 ± 0.02a	4.26 ± 0.11a	4.41 ± 0.15a
s_E1followedbysE2	4.94 ± 0.20a	4.15 ± 0.04b	4.73 ± 0.18a	4.98 ± 0.17a
t_endLpd.E1followedbysE2	6.60 ± 0.75a	5.30 ± 0.69a	6.05 ± 0.58a	3.91 ± 0.04a
rel_E1_E12	0.20 ± 0.02b	0.16 ± 0.06b	0.14 ± 0.01b	0.54 ± 0.19a
E2index	0.82 ± 0.13a	0.99 ± 0.15a	0.88 ± 0.13a	0.86 ± 0.19a
%sE2/E2	1.16 ± 0.16a	1.06 ± 0.19a	1.26 ± 0.11a	0.83 ± 0.24a

^1^ The values in the table show means ± stand error (SE). The data were compared using the analysis of variance (ANOVA) followed by the unrestricted least significant differences (LSD) after square-root transformation for frequency variables, natural log transformation for time variables, and square arcsine for percentage variables. The level for significance was set to *P* < 0.05. The values followed by the same lowercase letter within a row represent no significant differences among the four cultivars. The acronym of the variables in the first column was defined in Table 1.
